# Short- and Long-Term Prognosis of COVID-19-Associated Versus Non-COVID-19 Takotsubo Cardiomyopathy: A Propensity-Matched Nationwide Study

**DOI:** 10.7759/cureus.101631

**Published:** 2026-01-15

**Authors:** Didien Meyahnwi, Yussif Issaka, Efeturi M Okorigba, Vanessa O Agberien, Simran Joshi, Zachary Port

**Affiliations:** 1 Department of Medicine, Yale New Haven Health Bridgeport Hospital, Bridgeport, USA; 2 Department of Internal Medicine, West Virginia University, Morgantown, USA; 3 Department of Cardiology, Yale School of Medicine, New Haven, USA

**Keywords:** acute systolic heart failure, cardiac complications, covid-19, heart failure with reduced ejection fraction, long-covid-19, sars-cov-2 infection, stress induced cardiomyopathy, takotsubo cardiomyopathy

## Abstract

Background: Takotsubo cardiomyopathy (TCM) is a stress-induced cardiomyopathy with transient left ventricular dysfunction, often triggered by acute illness. Coronavirus disease 2019 (COVID-19) has been implicated as a precipitant, but comparative outcome data for COVID-19-related versus non-COVID-19 TCM remain largely limited to the early pandemic, when few effective treatments existed.

Methods: We conducted a retrospective cohort study using the TriNetX database, identifying US adults hospitalized between March 2020 and March 2025 with TCM and created two cohorts: patients with TCM and laboratory-confirmed COVID-19 and patients with TCM without COVID-19, both diagnosed within 14 days of admission. The primary outcome was 30-day all-cause mortality, with secondary 30-day outcomes and one-year mortality also assessed. Hazard ratios (HRs) with 95% confidence intervals (CIs) were estimated.

Results: Of 16,649 patients (6,367 COVID-19 TCM and 10,282 non-COVID-19 TCM), 5,955 per group remained after propensity score matching. Baseline demographics and comorbidities were well balanced (mean age 66.8 years; 76.8% female). Thirty-day mortality did not differ significantly between COVID-19 and non-COVID-19 TCM (12.0% vs. 11.1%; HR 1.07, 95% CI 0.97-1.19). However, COVID-19 TCM was associated with higher risks of heart failure with reduced ejection fraction (HFrEF) (HR 1.18, 95% CI 1.10-1.26), cardiogenic shock (HR 1.25, 95% CI 1.08-1.46), and ventricular arrhythmias (HR 1.37, 95% CI 1.14-1.64). One-year mortality was significantly higher in the COVID-19 cohort (22.0% vs. 18.3%; HR 1.19, 95% CI 1.10-1.29).

Conclusion: COVID-19-associated TCM is not linked to excess short-term mortality but carries higher risks of acute complications and significantly worse one-year survival, highlighting the persistent effects of COVID-19 after an acute infection.

## Introduction

Coronavirus disease 2019 (COVID-19), caused by the severe acute respiratory syndrome coronavirus-2 (SARS-CoV-2), was first identified in late 2019 and has since evolved into a global pandemic, contributing to millions of deaths worldwide [1-3]. In the United States alone, the Centers for Disease Control and Prevention (CDC) estimated that between October 2024 and August 2025, COVID-19 accounted for 38,000-57,000 deaths, underscoring its enduring role in human disease burden [4]. While the predominant clinical presentation of COVID-19 is severe pneumonia leading to acute respiratory distress syndrome (ARDS), respiratory failure, circulatory shock, and death [5], mounting evidence highlights significant cardiovascular involvement as well. COVID-19 has been associated with a spectrum of cardiac complications including myocarditis, arrhythmias, thromboembolic events, and cardiac arrest [6-8].

Among these manifestations, Takotsubo cardiomyopathy (TCM), a stress-induced cardiomyopathy mimicking acute coronary syndrome but characterized by transient left ventricular systolic dysfunction without obstructive coronary artery disease, has been previously described in patients with COVID-19 [9-14]. The pathophysiological mechanisms remain incompletely defined but are thought to mirror those of non-COVID-19 TCM, including catecholamine-induced myocardial stunning, systemic inflammation, cytokine storm, and microvascular dysfunction [11,12,15,16]. The cytokine storm and proinflammatory state during a bout of COVID-19 make patients with this disease prime subjects for TCM. Notably, those with severe COVID-19 often require catecholamine vasopressor therapy (e.g., norepinephrine and epinephrine), which, combined with the heightened proinflammatory milieu, could further contribute to myocardial stunning and dysfunction. In an analysis of the National Inpatient Sample (NIS), Davis and colleagues reported that COVID-19 patients who developed TCM experienced inpatient mortality rates exceeding twice those of COVID-19 patients without TCM (32.8% vs. 14.6%) [14]. Because of the underlying pathophysiology and available data, we therefore postulate that COVID-19 patients with TCM would have worse outcomes relative to TCM patients without COVID-19.

The majority of available evidence of COVID-19-associated TCM is limited to case reports, small case series, or single-center cohorts [11-13,15]. There are a few multicenter studies that compared outcomes of TCM in patients with COVID-19 to those without COVID-19 [17,18]. In one study (a systematic review), the authors reported mortality rates as high as 26% (20/77) among patients with COVID-19-associated TCM, compared with 5.6% (7/123) in TCM associated with other respiratory illnesses, and 4.2% (57/1,353) in TCM without respiratory disease [17]. A large study by Hajra et al. based on the NIS utilized data from the early days of the pandemic (2020) when effective treatments were not yet widely known and/or available, and reported an inpatient mortality rate of 33.9% in TCM patients with COVID-19 versus 7.3% in TCM patients without COVID-19 [18]. However, there are now well-established treatments and vaccines for COVID-19, viral variants have evolved, and supportive management has clearly improved from its early days, making that previous data unreliable.

Given this critical gap in literature and the persistent contribution of COVID-19 to human disease burden, we designed the present study to test the hypothesis that COVID-19-associated TCM is linked to worse short-term outcomes (30-day mortality and acute complications including need for mechanical ventilation, cardiac vasopressors, cardiac mechanical circulatory support, etc.) compared with non-COVID-19 TCM. One-year mortality post TCM episode would be assessed for any persistent difference (if any) in mortality risk after acute hospitalization. By leveraging TriNetX, a large, geographically diverse, real-world clinical database encompassing over 60 US healthcare organizations (HCOs), we sought to provide robust contemporary evidence regarding 30-day mortality and complications in this population.

## Materials and methods

We performed a retrospective cohort study using the TriNetX US Collaborative Network, which aggregates electronic health record (EHR) data from multiple HCOs across the United States, covering over 110 million patients. We queried data from 39 HCOs, including adult patients (≥18 years) hospitalized between March 1, 2020, and March 1, 2025, with a diagnosis of TCM.

Cohort definitions

**Figure 1:**
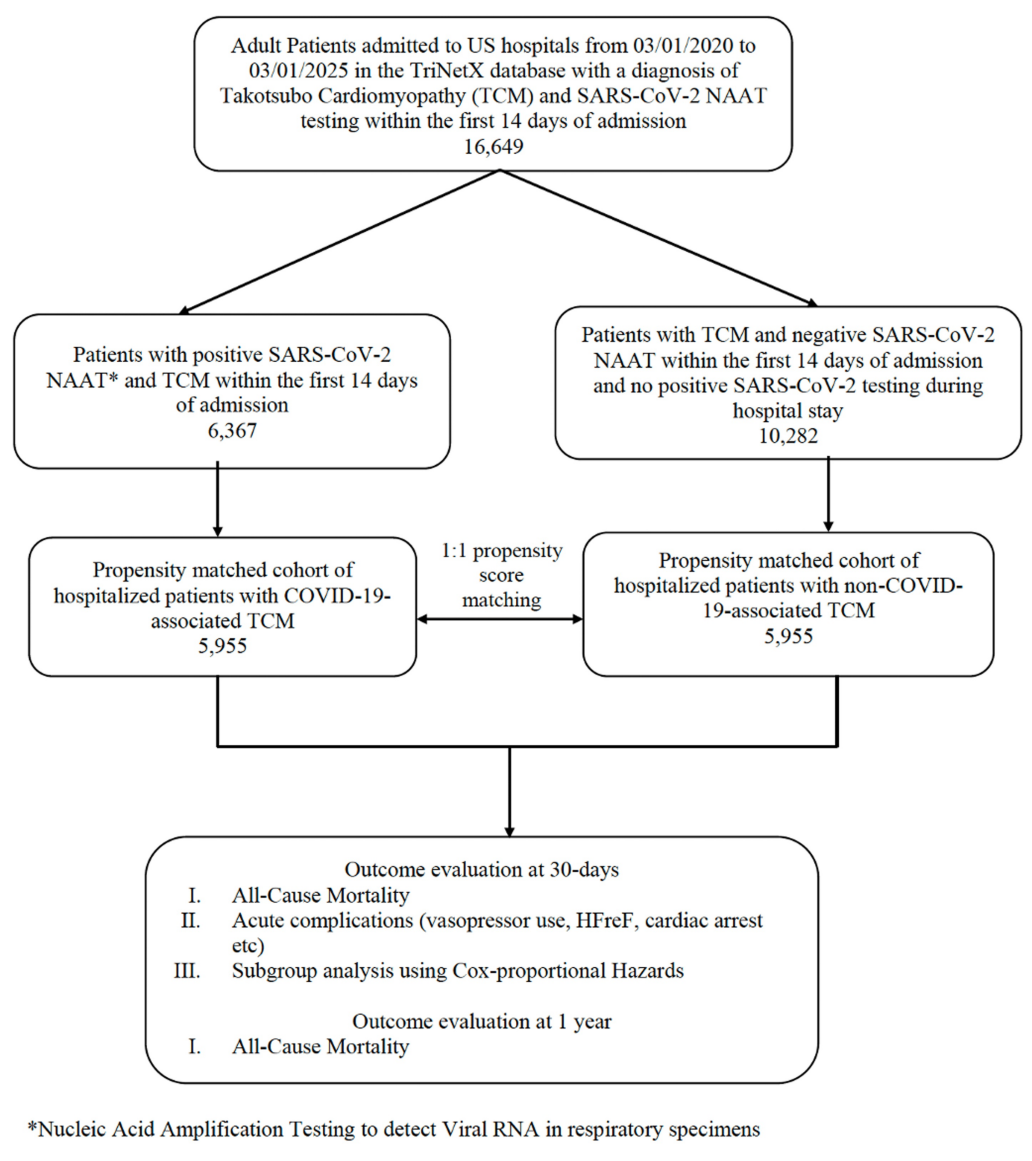
Methodology Flowchart and Cohort Selection SARS-CoV-2: severe acute respiratory syndrome coronavirus-2; COVID-19: coronavirus disease 2019; HFrEF: heart failure with reduced ejection fraction

Two mutually exclusive cohorts were defined: (1) patients admitted to the hospital with laboratory-confirmed SARS-CoV-2 infection (by nucleic acid amplification testing (NAAT) on a respiratory specimen) and a diagnosis of TCM within the first 14 days of admission (COVID-19-associated TCM) and (2) patients admitted with TCM but with negative NAAT for SARS-CoV-2 RNA during the entire hospitalization course (non-COVID-19 TCM). The index date was defined as the first date meeting the cohort eligibility criteria. This design reduces the likelihood of incidental or hospital-acquired infection being misclassified as COVID-19-associated TCM. To reduce confounding, 1:1 propensity score matching (PSM) was performed between the COVID-19 and non-COVID-19 cohorts. Matching was rigorous and based on demographics, relevant comorbidities, and select medication exposures, all specified in Table 1, using a nearest-neighbor algorithm without replacement and a caliper of 0.1 of the pooled standard deviation of the logit of the propensity score. Covariate balance was assessed using standardized mean differences (SMDs), with SMD < 0.1 indicating acceptable balance (Figure 1).

**Table 1:** Baseline Characteristics of Patients With Takotsubo Cardiomyopathy, Before and After Propensity Score Matching COVID-19: coronavirus disease 2019; TCM: Takotsubo cardiomyopathy; SD: standard deviation; COPD: chronic obstructive pulmonary disease; CKD: chronic kidney disease; RAAS: renin-angiotensin-aldosterone system

	Before matching				After matching			
Characteristic	COVID-19 TCM (n = 6,367)	Non–COVID-19 TCM (n = 10,282)	p-value	Std Diff	COVID-19 TCM (matched, n = 5,955)	Non-COVID-19 TCM (matched, n = 5,955)	p-value	Std Diff
Age								
Age, mean ± SD	66.9 ± 15.0	66.4 ± 15.1	0.027	0.035	66.8 ± 15.2	66.8 ± 15.0	0.962	0.001
Race/ethnicity, n (%)								
Female sex	4,914 (77.2)	7,818 (76.0)	0.091	0.027	4,571 (76.8)	4,571 (76.8)	1.000	<0.001
Male sex	1,453 (22.8)	2,461 (23.9)	0.099	0.026	1,384 (23.2)	1,383 (23.2)	0.983	<0.001
White	5,030 (79.0)	7,831 (76.2)	<0.001	0.068	4,702 (79.0)	4,712 (79.1)	0.822	0.004
Black/African American	556 (8.7)	1,218 (11.8)	<0.001	0.103	552 (9.3)	567 (9.5)	0.638	0.009
Asian	284 (4.5)	279 (2.7)	<0.001	0.094	223 (3.7)	231 (3.9)	0.702	0.007
Hispanic/Latino	326 (5.1)	584 (5.7)	0.123	0.025	317 (5.3)	305 (5.1)	0.621	0.009
Not Hispanic/Latino	5,419 (85.1)	8,385 (81.6)	<0.001	0.096	5,040 (84.6)	5,106 (85.7)	0.089	0.031
Comorbidities, n (%)								
Hypertension	4,093 (64.3)	5,557 (54.0)	<0.001	0.209	3,766 (63.2)	3,743 (62.9)	0.662	0.008
Diabetes mellitus	1,853 (29.1)	2,374 (23.1)	<0.001	0.137	1,680 (28.2)	1,663 (27.9)	0.729	0.006
Hyperlipidemia	3,126 (49.1)	4,063 (39.5)	<0.001	0.194	2,833 (47.6)	2,836 (47.6)	0.956	0.001
Ischemic heart disease	3,323 (52.2)	3,811 (37.1)	<0.001	0.308	2,939 (49.4)	2,952 (49.6)	0.812	0.004
Neoplasms	2,677 (42.0)	3,505 (34.1)	<0.001	0.164	2,409 (40.5)	2,378 (39.9)	0.562	0.011
COPD	1,640 (25.8)	1,757 (17.1)	<0.001	0.212	1,401 (23.5)	1,407 (23.6)	0.897	0.002
Alcohol use disorder	680 (10.7)	828 (8.1)	<0.001	0.090	619 (10.4)	615 (10.3)	0.904	0.002
Tobacco use	585 (9.2)	732 (7.1)	<0.001	0.076	524 (8.8)	496 (8.3)	0.359	0.017
Acute myocardial infarction	2,180 (34.2)	2,153 (20.9)	<0.001	0.301	1,837 (30.8)	1,853 (31.1)	0.751	0.006
Atrial fibrillation/flutter	1,343 (21.1)	1,494 (14.5)	<0.001	0.172	1,165 (19.6)	1,174 (19.7)	0.836	0.004
Iron deficiency anemia	1,062 (16.7)	1,272 (12.4)	<0.001	0.123	944 (15.9)	940 (15.8)	0.920	0.002
CKD stage 3	910 (14.3)	1,066 (10.4)	<0.001	0.120	808 (13.6)	807 (13.6)	0.979	<0.001
CKD stage 4	261 (4.1)	295 (2.9)	<0.001	0.067	233 (3.9)	227 (3.8)	0.775	0.005
CKD stage 5	101 (1.6)	102 (1.0)	0.001	0.053	78 (1.3)	89 (1.5)	0.391	0.016
Obesity	1,214 (19.1)	1,542 (15.0)	<0.001	0.108	1,111 (18.7)	1,084 (18.2)	0.523	0.012
Hyperthyroidism	207 (3.3)	243 (2.4)	0.001	0.054	178 (3.0)	183 (3.1)	0.789	0.005
Medications, n (%)								
Diuretics	3,410 (53.6)	3,884 (37.8)	<0.001	0.321	3,027 (50.8)	3,016 (50.6)	0.840	0.004
Beta-blockers	3,820 (60.0)	4,811 (46.8)	<0.001	0.267	3,447 (57.9)	3,446 (57.9)	0.985	<0.001
Calcium channel blockers	2,482 (39.0)	3,160 (30.7)	<0.001	0.174	2,251 (37.8)	2,243 (37.7)	0.880	0.003
RAAS inhibitors	3,187 (50.1)	4,076 (39.6)	<0.001	0.211	2,892 (48.6)	2,894 (48.6)	0.971	0.001
Factor Xa inhibitors	1,179 (18.5)	1,362 (13.2)	<0.001	0.145	1,043 (17.5)	1,055 (17.7)	0.773	0.005
Warfarin	432 (6.8)	571 (5.6)	0.001	0.051	408 (6.9)	422 (7.1)	0.614	0.009
Aspirin	3,472 (54.5)	3,978 (38.7)	<0.001	0.322	3,097 (52.0)	3,118 (52.4)	0.700	0.007
Statins	3,380 (53.1)	4,359 (42.4)	<0.001	0.215	3,062 (51.4)	3,041 (51.1)	0.700	0.007
PCSK9 inhibitors	39 (0.6)	58 (0.6)	0.566	0.009	35 (0.6)	41 (0.7)	0.683	0.007
Ezetimibe	225 (3.5)	316 (3.1)	0.103	0.026	212 (3.6)	217 (3.6)	0.806	0.005
Laboratory values								
Hemoglobin, g/dL (mean ± SD)	11.8 ± 2.3 (5,336)	12.2 ± 2.2 (7,195)	<0.001	0.163	11.8 ± 2.3 (4,925)	12.1 ± 2.2 (4,868)	<0.001	0.101

Outcomes

The primary outcome was 30-day all-cause mortality. The secondary outcomes included new-onset heart failure with reduced ejection fraction (HFrEF), cardiogenic shock, extracorporeal membrane oxygenation (ECMO) use, life-threatening ventricular arrhythmias (composite of ventricular tachycardia and fibrillation/flutter), cardiac arrest, mechanical circulatory support (left ventricular assist device (LVAD) or intra-aortic balloon pump), invasive or non-invasive positive pressure ventilation, and cardiac pressor therapy (dobutamine, epinephrine, norepinephrine, milrinone, or dopamine). For non-fatal outcomes, patients were considered at risk from day 1 after the index event, which was defined as cohort eligibility. Secondary outcomes were exploratory, with no multiplicity adjustment. To assess for lingering effects of COVID-19-associated TCM versus non-COVID-19 TCM, mortality was assessed again at one year post-index event. All ICD-10 (International Classification of Diseases, 10th Revision) diagnostic and procedure codes used to define the cohort and outcome variables are listed in Table 2 (Figure 1).

**Table 2:** ICD-10 Codes Used to Define the Cohort and Outcomes CPT: Current Procedural Terminology; LNC: Logical Observation Identifier Names and Codes; HFrEF: heart failure with reduced ejection fraction; ECMO: extracorporeal membrane oxygenation; SARS-CoV-2: severe acute respiratory syndrome coronavirus-2; COVID-19: coronavirus disease 2019

Outcome	ICD-10 code/CPT code
HFrEF	I50.2
Cardiogenic shock	R57.0
Non-invasive or invasive positive pressure ventilation	5A1935Z, 5A1945Z, 5A1955Z, CPT: 1014859, CPT: 94002, LNC: 20124-4, CPT: 94003, Z99.11, Z99.89, 5A09357, 5A09457, 5A09557, LNC:58959-8
Cardiac arrest	I46
Mechanical circulatory support	Z95.811, 5A02210, 5A0221D, 02HA3RJ, 02HA3RZ
Life-threatening ventricular arrhythmias	I47.2, I49.0
Cardiac pressors	dobutamine (RXNORM: 3616), epinephrine (NORM: 3992), norepinephrine (RXNORM: 7512), milrinone (RXNORM: 52769), dopamine (RXNORM: 3628)
ECMO	CPT: 1021846, 5A15223, 5A1522F, 5A1522G, 5A1522H
Takotsubo cardiomyopathy	I51.81
SARS-CoV-2 (COVID-19) RNA	LNC: 94500-6

Statistical analysis

Continuous variables were reported as mean ± standard deviation, and categorical variables were expressed as counts and percentages. Survival analyses were performed with Kaplan-Meier curves and log-rank testing. Effect estimates were expressed as risk ratios (RRs), derived from cumulative incidence, and hazard ratios (HRs) with 95% confidence intervals (CIs). Patients were censored at outcome occurrence, death, last clinical encounter, or end of follow-up window. For missing data, no imputation was done. Analyses were performed using available data for each outcome.

To evaluate for effect heterogeneity across different patient characteristics, stratified analyses were performed across different subgroups. Subgroup analyses were conducted within the propensity-matched cohort. The subgroups included age (18-64 or ≥65 years), sex, ethnicity, atrial fibrillation/flutter, estimated glomerular filtration rate (eGFR) (mL/min/1.73 m²), essential hypertension, history of ischemic cardiomyopathy, and pre-existing heart failure. The subgroup analysis was conducted using a Cox proportional hazards model, creating subsets of patients from the group used in the primary outcome analysis. All analyses were conducted within the TriNetX platform, with a two-sided p-value < 0.05 considered statistically significant. Because the database is de-identified, this study was exempt from institutional review board approval (Figure 1).

## Results

A total of 16,649 patients were identified (6,367 with COVID-19-associated TCM and 10,282 with non-COVID-19 TCM). After 1:1 PSM, 5,955 patients remained in each group with well-balanced baseline characteristics (mean age 66.8 ± 15.2 vs. 66.8 ± 15.0 years; 76.8% vs. 76.8% female; SMDs < 0.1 across variables). Matching retained 93.5% of COVID-19 TCM and 57.9% of non-COVID-19 TCM patients. See Table 1 for all baseline characteristics.

Primary outcome

**Figure 2:**
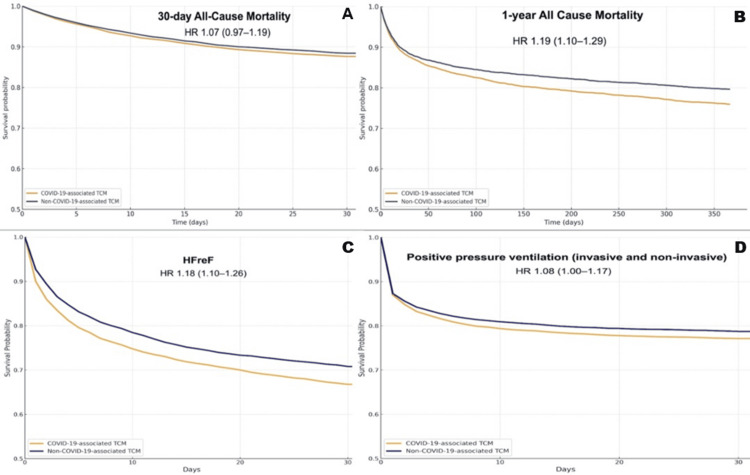
(A-D) Kaplan-Meier Curves for the Primary Outcome Variable and Select Secondary Outcomes HR: hazard ratio; COVID-19: coronavirus disease 2019; TCM: Takotsubo cardiomyopathy; HFrEF: heart failure with reduced ejection fraction

Thirty-day mortality was not significantly different between COVID-19-associated and non-COVID-19 TCM (12.0% vs. 11.1%; RR 1.1, 95% CI 0.97-1.19). In the time-to-event analyses, Kaplan-Meier curves confirmed similar overlapping survival probabilities (87.6% vs. 88.4%), and the HR for death in the COVID-19 cohort was 1.07 (95% CI 0.97-1.19; p = 0.65) (Figure 2).

At one year, mortality was significantly higher among patients with COVID-19-associated TCM (22.0% vs. 18.3%, RR 1.20, 95% CI 1.12-1.29). Kaplan-Meier survival analysis demonstrated reduced survival probability at one year in the COVID-19 + TCM cohort (75.9% vs. 79.6%), with a 19% increased hazard of death at one year (HR 1.19, 95% CI 1.10-1.29) (Figure 2).

Secondary outcomes

Patients with COVID-19-associated TCM experienced significantly higher rates of new or worsening HFrEF (31.9% vs. 27.9%; HR 1.18, 95% CI 1.10-1.26; p < 0.001). Cardiogenic shock was also more common in the COVID-19 group (6.3% vs. 5.0%; HR 1.25, 95% CI 1.08-1.46; p = 0.004). The requirement for non-invasive or invasive positive pressure ventilation was elevated in the COVID-19 group (22.5% vs. 20.9%; HR 1.08, 95% CI 1.00-1.17; p = 0.04). Rates of cardiac arrest were similar between both cohorts (4.3% vs. 3.7%; HR 1.16, 95% CI 0.97-1.39; p = 0.09). Mechanical circulatory support, although infrequent overall, was statistically more frequent among COVID-19 patients (1.2% vs. 0.7%; HR 1.57, 95% CI 1.08-2.29; p = 0.02). Similarly, life-threatening ventricular arrhythmias occurred more commonly in the COVID-19 group (4.7% vs. 3.4%; HR 1.37, 95% CI 1.14-1.64; p = 0.001) (Table 3). The ICD-10 codes used to define these secondary outcomes are listed in Table 2. These differences persisted at the one-year mark, as shown in Table 4.

**Table 3:** Thirty-Day Outcomes and 1-Year All-Cause Mortality in COVID-19-Associated vs. Non-COVID-19 Takotsubo Cardiomyopathy (After Propensity Score Matching, n = 5,955 per group) COVID-19: coronavirus disease 2019; TCM: Takotsubo cardiomyopathy; CI: confidence interval; HFrEF: heart failure with reduced ejection fraction; ECMO: extracorporeal membrane oxygenation

Outcome	Risk in COVID-19 TCM (N = 5,955) n (%)	Risk in non-COVID-19 TCM (N = 5,955) n (%)	Relative risk (95% CI)	Hazard ratio (95% CI)
30-day all-cause mortality	713 (12.0)	663 (11.1)	1.08 (0.97–1.19)	1.07 (0.97–1.19)
HFrEF	1,900 (31.9)	1,659 (27.9)	1.15 (1.08–1.21)	1.18 (1.10–1.26)
Cardiogenic shock	374 (6.3)	300/5,955	1.25 (1.08–1.45)	1.25 (1.08–1.46)
Positive pressure ventilation	1,338 (22.5)	1,245 (20.9)	1.08 (1.00–1.15)	1.08 (1.00–1.17)
Cardiac arrest	259 (4.3)	223 (3.7)	1.16 (0.98–1.38)	1.16 (0.97–1.39
Mechanical circulatory support	69 (1.2)	44 (0.7)	1.57 (1.08–2.29)	1.57 (1.08–2.29)
Life-threatening ventricular arrhythmia	277 (4.7)	203 (3.4)	1.37 (1.14–1.63)	1.37 (1.14–1.64)
Cardiac pressors	1,591 (26.7)	1,611 (27.1)	0.99 (0.93–1.05)	0.99 (0.92–1.06)
ECMO (unnamed outcome)	24 (0.4)	23 (0.4)	1.04 (0.59–1.85)	1.04 (0.59–1.85)
1-year all-cause mortality	1,311 (22.0)	1,092 (18.3)	1.20 (1.12–1.29)	1.19 (1.10–1.29)

**Table 4:** One-Year Outcomes in Patients With COVID-19-Associated vs. Non-COVID-19 Takotsubo Cardiomyopathy HFrEF: heart failure with reduced ejection fraction; ECMO: extracorporeal membrane oxygenation; COVID-19: coronavirus disease 2019; TCM: Takotsubo cardiomyopathy; CI: confidence interval

Outcome	COVID-19 TCM (n = 5,955)	Non-COVID-19 TCM (n = 5,955)	Relative risk (95% CI)	Hazard ratio (95% CI)
All-cause mortality	1,311 (22.0)	1,092 (18.3)	1.20 (1.12–1.29)	1.19 (1.10–1.29)
HFrEF	2,430 (40.8)	2,173 (36.5)	1.12 (1.07–1.17)	1.15 (1.09–1.22)
Cardiogenic shock	449 (7.5)	360 (6.0)	1.25 (1.09–1.43)	1.25 (1.09–1.44)
Non-invasive or invasive ventilation	1,649 (27.7)	1,450 (24.3)	1.14 (1.07–1.21)	1.15 (1.07–1.23)
Cardiac arrest	350 (5.9)	297 (5.0)	1.18 (1.01–1.37)	1.18 (1.01–1.37)
Mechanical circulatory support	84 (1.4)	51 (0.9)	1.65 (1.17–2.33)	1.64 (1.16–2.33)
Life-threatening ventricular arrhythmia	402 (6.8)	325 (5.5)	1.24 (1.07–1.43)	1.24 (1.07–1.43)
Cardiac pressors	2,052 (34.5)	1,994 (33.5)	1.03 (0.98–1.08)	1.03 (0.97–1.10)
ECMO	25 (0.4)	28 (0.5)	0.89 (0.52–1.53)	0.89 (0.52–1.53)

In contrast, the use of intravenous cardiac pressors (26.7% vs. 27.1%; HR 0.99, 95% CI 0.92-1.06; p = 0.72) and extracorporeal membrane oxygenation (0.4% vs. 0.4%; HR 1.04, 95% CI 0.59-1.85; p = 0.57) did not differ significantly between the two groups (Table 3).

Cox proportional hazards model

**Figure 3:**
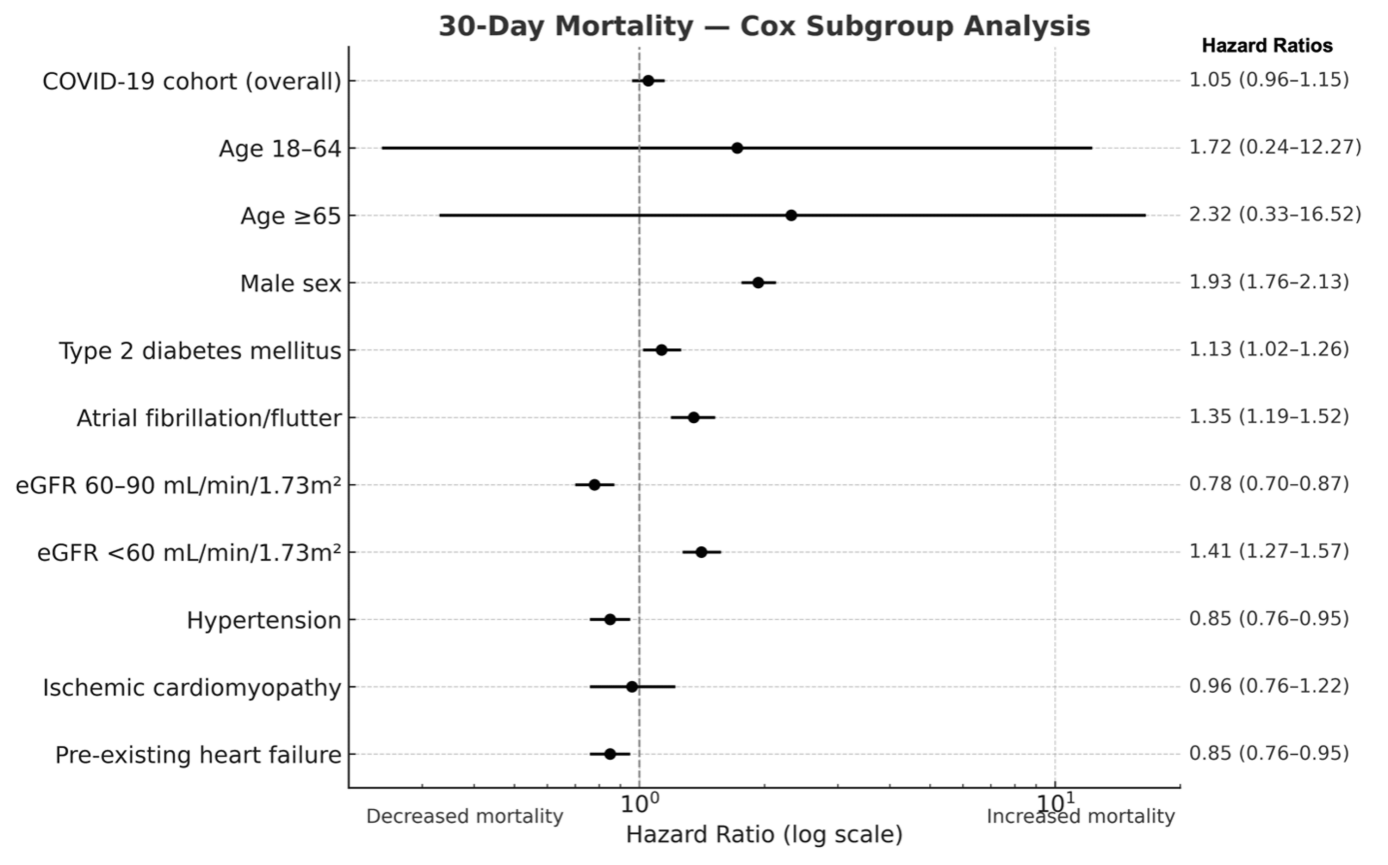
Thirty-Day Mortality Across Different Subgroups Using the Cox Proportional Hazards Model eGFR: estimated glomerular filtration rate; COVID-19: coronavirus disease 2019

Though in the overall cohort, 30-day mortality did not differ between the two groups, the multivariable Cox regression showed that certain subgroups with COVID-19-associated TCM had increased 30-day mortality. Male sex conferred nearly a twofold higher hazard of death (HR 1.93, 95% CI 1.76-2.13; p < 0.001). Type 2 diabetes mellitus had increased mortality hazards (HR 1.13, 95% CI 1.02-1.26; p = 0.02), as did atrial fibrillation/flutter (HR 1.35, 95% CI 1.19-1.52; p < 0.001). Those with eGFR of at least 60 mL/min/1.73m² had lower hazard of death (HR 0.78, 95% CI 0.70-0.87; p < 0.001), whereas those with eGFR < 60 mL/min/1.73 m²) had higher mortality hazard (HR 1.41, 95% CI 1.27-1.57; p < 0.001) (Figure 3).

Pre-existing hypertension (HR 0.85, 95% CI 0.76-0.95; p = 0.004) and pre-existing heart failure (HR 0.85, 95% CI 0.76-0.95; p = 0.004) were paradoxically associated with lower 30-day mortality. On the other hand, pre-existing ischemic cardiomyopathy was not significantly associated with 30-day mortality (HR 0.96, 95% CI 0.76-1.22; p = 0.74) (Figure 3).

## Discussion

In this large, nationwide analysis of hospitalized patients with TCM, we observed several key findings. First, short-term (30-day) mortality was not significantly different between COVID-19-associated TCM and non-COVID-19 TCM, with survival probabilities largely overlapping in early follow-up. However, patients with COVID-19-associated TCM experienced higher rates of acute complications, including new or worsening heart failure, cardiogenic shock, ventricular arrhythmias, and need for mechanical circulatory support. Importantly, one-year follow-up revealed a significant survival disadvantage in the COVID-19 cohort, with a 19% higher hazard of death compared to non-COVID-19 counterparts. Subgroup analyses showed that certain patient characteristics, including age ≥ 65 years, male sex, diabetes mellitus, atrial fibrillation/flutter, and impaired renal function, conferred higher mortality risk in the COVID-19-associated TCM relative to their counterparts with non-COVID-19 TCM, while hypertension and pre-existing heart failure were paradoxically associated with improved short-term outcomes.

Our results extend prior observations of TCM in the setting of COVID-19, which have largely been restricted to case reports, case series, and single-center studies [7-11]. In contrast to an earlier study suggesting dramatically higher in-hospital mortality among COVID-19 TCM patients relative to non-COVID-19 patients, our nationwide analysis demonstrated that 30-day mortality was only modestly elevated and did not achieve statistical significance [18]. Several explanations may account for this divergence. First, improvements in inpatient management of COVID-19 since the early phases of the pandemic, including standardized use of corticosteroids, anticoagulation strategies, antivirals, and refined ventilatory practices, may have mitigated early excess mortality risk in this population. Second, vaccines became more widely available, which could have contributed to decreased morbidity and mortality in COVID-19 TCM patients. Third, patient populations studied are not uniform. Our study limits inclusion to patients who received a diagnosis of TCM ± COVID-19 within the first 14 days of admission, increasing the probability that these are the pathologies driving the admission. The study by Hajra et al., however, does not put a time constraint on when participants were diagnosed with the conditions during admission, increasing cohort heterogeneity, potentially picking up high acuity patients with hospital-acquired COVID-19, and skewing results [18].

Despite similar early survival, the elevated one-year mortality among COVID-19-associated TCM patients deserves emphasis. This aligns with findings by Xie and colleagues who reported that beyond the first 30 days of COVID-19 infection, relative to non-COVID-19-infected peers, patients with previous COVID-19 infection are at increased risk of several cardiovascular disorders including ischemic and non-ischemic heart disease, heart failure, thromboembolic disease, and dysrhythmias, which puts them at increased risk of death [19]. This has been corroborated by several longitudinal studies of COVID-19 survivors (beyond the acute COVID-19 episode) that have consistently shown persistent inflammation, endothelial dysfunction, and increased thrombotic risk up to even a year after infection [19-22]. For example, Puntmann and colleagues, following COVID-19 survivors for a median of 71 (64-92) days post COVID-19 diagnosis, showed that compared to healthy and risk factor matched controls, COVID-19 survivors had lower left ventricular ejection fraction, increased troponins, inflammatory changes on cardiac MRI, and active lymphocytic inflammation in endomyocardial biopsy in patients with severe inflammation [20]. These sequelae may synergize with the myocardial stunning and autonomic dysregulation inherent to TCM, predisposing patients to recurrent decompensation or progressive cardiomyopathy. This implies that long-term outcomes in COVID-19-associated TCM stem less from TCM itself and more from its interaction with the enduring cardiometabolic impact of COVID-19.

The higher rates of secondary complications in COVID-19-associated TCM further support the hypothesis that COVID-19 potentiates cardiovascular instability. Our observation of a greater risk of cardiogenic shock, ventricular arrhythmias, and need for mechanical support parallels prior studies demonstrating heightened catecholamine exposure, cytokine-mediated myocardial injury, and microvascular dysfunction in COVID-19 patients [23]. Notably, the increased incidence of life-threatening arrhythmias may reflect synergistic effects of viral myocarditis, systemic inflammation, and, to a lesser extent, QT-prolonging therapies used early in the pandemic. The fact that cardiac arrest did not differ significantly between cohorts suggests that rapid recognition and intervention may have averted progression to terminal events, even as complications were more common in the COVID-19 group.

The subgroup analyses provide further clinical insight. Male sex nearly doubled mortality risk, consistent with both COVID-19 outcome studies and non-COVID-19 TCM epidemiology, where women typically have more favorable survival [24,25]. Similarly, comorbid diabetes and atrial fibrillation conferred additive mortality hazards, reflecting their known association with impaired myocardial recovery and higher arrhythmogenic potential [26,27]. Renal dysfunction emerged as a particularly strong predictor, highlighting the interplay of multiorgan injury in kidney disease, COVID-19, and TCM, and consistent with data previously from a Spanish registry showing worse outcomes in patients with TCM who had concurrent renal impairment (acute or chronic) [28]. The decreased mortality hazard observed with hypertension and pre-existing heart failure was unexpected, potentially in part due to earlier hospital presentation, higher healthcare utilization, and heightened surveillance. We also suggest these patients are more likely to be on some component of Guideline-Directed Medical Therapy for Heart Failure (GDMT), which helps suppress the deleterious neurohormonal cycle in heart failure and prevent cardiac remodeling, thereby improving hemodynamic resilience and improving survival. It is also possible that this paradoxical association was driven by survivor bias. These nuanced findings underscore the heterogeneity of outcomes in TCM and the importance of individualized risk stratification.

Strengths and limitations

The strengths of our study include the use of a large, contemporary, geographically diverse, real-world dataset encompassing almost 40 HCOs across the United States, enabling a comprehensive, up-to-date, comparative evaluation of COVID-19-associated versus non-COVID-19 TCM outcomes. Rigorous PSM minimized confounding, and the inclusion of long-term mortality and complications, in addition to short-term outcomes, allowed for a more complete understanding of the clinical trajectory of these patients.

Nevertheless, several limitations should be acknowledged. First, as with all retrospective analyses of EHRs, coding inaccuracies and misclassification bias are possible. Notably, because TriNetX does not allow adjudication of TCM using InterTAK criteria, we acknowledge potential misclassification and have provided the full code lists in Table 2. Second, though we required SARS-CoV-2 NAAT positivity and TCM diagnosis to occur within the first 14 days of admission, this temporal restriction strengthens the association but does not establish causality or rule out incidental infection. Third, residual confounding by unmeasured variables cannot be excluded despite rigorous PSM. In addition, other balancing methods such as inverse probability treatment weighting could not be performed within our analysis platform. Fourth, not all TriNetX member organizations report granular echocardiographic or biomarker data, precluding assessment of inflammatory burden, left ventricular dysfunction, and recovery. Fifth, the severity of COVID-19 infection (with granular details such as oxygen requirements and ICU-level physiologic scores), vaccination status, viral variants, and treatment strategies were not uniformly available, potentially creating heterogeneity in our cohort, and while mortality was assessed through robust linkage, cause-specific mortality could not be reliably distinguished. Sixth, competing risks were not formally modeled, and proportional hazards assumptions and collinearity could not be directly tested within TriNetX.

## Conclusions

In this large, nationwide matched analysis, we found that COVID-19-associated TCM was not associated with higher 30-day mortality compared with non-COVID-19 TCM but was characterized by increased acute complications and a significant survival disadvantage at one year. Male sex, diabetes, atrial fibrillation/flutter, and renal dysfunction were associated with worse 30-day mortality in the COVID-19-positive versus non-COVID-19 TCM cohort, whereas hypertension and pre-existing heart failure were paradoxically associated with lower short-term mortality. These findings highlight the need for vigilant long-term follow-up and comprehensive cardiovascular risk management in patients with COVID-19-associated TCM and align with growing literature documenting higher long-term cardiovascular morbidity and mortality among post-COVID-19 populations. Future research should aim to develop targeted strategies for surveillance and intervention in this high-risk population.
